# Immune Modulatory Potential of Anti-idiotype Antibodies as a Surrogate of Foot-and-Mouth Disease Virus Antigen

**DOI:** 10.1128/mSphere.00522-18

**Published:** 2018-10-17

**Authors:** Ahsan Naveed, Sajjad Ur Rahman, Muhammad Imran Arshad, Bilal Aslam

**Affiliations:** a Institute of Microbiology, University of Agriculture, Faisalabad, Pakistan; b Institute of Pharmacy Physiology and Pharmacology, University of Agriculture, Faisalabad, Pakistan; University of Maryland, College Park

**Keywords:** anti-idiotype, antibody titer, foot-and-mouth disease, vaccine

## Abstract

The immunoprophylactic potential of anti-idiotype (anti-id) foot-and-mouth disease (FMD) antigen (Ag) was evaluated in the calves. The idiotype antibodies (Ab1) were produced in experimental goats by injecting inactivated FMD virus. The Fab (fragment antigen binding) of Ab1 was injected into the layer birds to raise anti-id antibodies (Ab2). The Ab2 was purified from egg yolks. The Fab component of Ab2 was emulsified in Montanide (1:1) and used as a surrogate of FMD virus. The immune response to Montanide adjuvanted monovalent and trivalent anti-id FMD virus antigen was determined in mice. The comparative immune potentiation potentials of Montanide adjuvanted trivalent anti-id FMD virus antigen and trivalent FMD vaccine were determined in mice and calves. Montanide adjuvanted monovalent anti-id FMD virus antigens produced mean Ab titers of 78.80%, 81.30%, and 81.20% for serotypes A, Asia 1, and O, respectively, at 45 days postimmunization (p.i.) in mice. Montanide adjuvanted trivalent anti-id FMD Ag in mice produced the highest Ab titer, 81.60%, at day 45 compared to the 77.50% titer measured for Montanide adjuvanted FMD vaccine at day 45 p.i. A slow decrease of 1% to 2% was recorded for the Ab titer of Montanide adjuvanted trivalent anti-id FMD virus antigen in mice at day 60. In calves, the titer corresponding to the immune response seen with Montanide adjuvanted trivalent anti-id FMD virus antigen (80%) was persistent whereas the titer of Montanide adjuvanted FMD vaccine decreased to 74% at day 60 p.i. Anti-id FMD virus antigen induced a strong and persistent immunogenic response in terms of Ab titer compared to the inactivated virus vaccine. Anti-id FMD virus antigen may serve as a surrogate of FMD virus vaccine.

**IMPORTANCE** Foot-and-mouth disease (FMD) is a contagious viral disease of animals. Multiple serotypes and antigenic variation in the viral genome are probably the factors that reduce control of the disease. Currently, the vaccines employed against FMD use killed virus. The inactivation or killing of the virus makes it less immunogenic and reduces its immunoprophylactic potential. To cope with this situation, the present study was designed, anti-idiotype FMD virus antigen was prepared, and the immunogenic potential of the antigen was compared to that of commercial killed-virus vaccines. The overall results showed that a persistent and strong immune response occurred with anti-idiotype FMD virus antigen. Thus, anti-idiotype FMD virus antigen may serve as a potential surrogate of FMD virus vaccines.

## Abstract (precis)

Foot-and-mouth disease (FMD) is a contagious viral disease of animals. Multiple serotypes and antigenic variation in the viral genome are probably the factors that reduce control of the disease. Currently, the vaccines employed against FMD use killed virus. The inactivation or killing of the virus makes it less immunogenic and reduces its immunoprophylactic potential. To cope with this situation, the present study was designed, anti-idiotype FMD virus antigen was prepared, and the immunogenic potential of the antigen was compared to that of commercial killed-virus vaccines. The overall results showed that a persistent and strong immune response occurred with anti-idiotype FMD virus antigen. Thus, anti-idiotype FMD virus antigen may serve as a potential surrogate of FMD virus vaccines.

## INTRODUCTION

The disease of animals that is most contagious and endemic in most countries is caused by members of the genus *Aphthovirus* of family *Picornaviridae*. Foot-and-mouth disease (FMD) virus contains a genome of 8.5 kb; the open reading frame (ORF) of the virus is translated into a polyprotein that, on posttranscriptional modification, produces four structural and eight nonstructural proteins (1). The virus has seven distinct serotypes, *viz*., Asia 1, A, O, C, SAT1, SAT2, and SAT3. The disease is most devastating since the virus has a broad host range. FMD virus affects all cloven-hooved animals and exhibits great antigenic variation (2). Upon infection of the host, the virus-associated molecular patterns are recognized by the recognition receptors in the host cell. The recognition of virus-associated molecular receptors causes interferon type 1 (type 1 IFN) and interferon-stimulated genes (ISGs) to be transcribed (3, 4). The downregulation of interferons is a stimulatory factor for enhanced expression of natural killer cells; thus, an antiviral state is established in the host (5). Serotypes Asia 1, A, and O target the livestock sector in Pakistan. Among the serotypes, Asia 1 and A are relatively stable whereas serotype O is unstable. The outbreaks associated with FMD virus are mainly tackled through vaccination. In conventional vaccines, an inactivated virus particle is used as an antigen. The virus becomes more unstable and less immunogenic upon inactivation. Furthermore, it becomes more expensive to maintain the integrity of the vaccine through the cold chain and frequent vaccination (6). There is risk associated with the virus inactivation process, since the genome may not completely inactivate or some virus particle may escape during inactivation.

Another approach to evoke an effective immune response is through the use of anti-idiotype (anti-id/Ab2) vaccines. Anti-id vaccines represent alternatives to protein- and peptide-based vaccines. Anti-id vaccines are generated against the idiotype of the primary antibodies (Ab1). The use of anti-id vaccines has been successfully practiced against various types of the human and animal antigens (7). Ab2 generated against outer membrane proteins (OMPs) of Pasteurella multocida provided 100% protection in immunized experimental animals. The anti-id vaccines have the ability to produce high and persistent antibody (Ab) titers (8). Anti-id vaccines induce not only humoral but also cellular immune responses and may be used as surrogate antigens (9). The current study aimed at developing Ab2 against FMD virus. Ab2 was evaluated for its immune potentiation potential in the natural host compared to commercially available killed-virus vaccines.

## RESULTS

Anti-id FMD antibodies were prepared in the layer birds. The development of Ab2 in the egg yolks of layer birds at day 14 postimmunization (p.i.) was confirmed through the use of the agar gel immunoprecipitation test (AGPT).

### Purification of anti-id antibodies.

The primary treatment of diluted egg yolk with ammonium sulfate (AS) resulted in yellow pellet formation upon centrifugation. More lipid contents were observed in primary treatment. The secondary treatment with 2 M AS resulted in precipitation of IgY proteins and in the formation of a clear white pellet. The IgY protein concentrations obtained from the egg yolks were more than 10 mg/ml in most of the samples processed.

**FIG 1:**
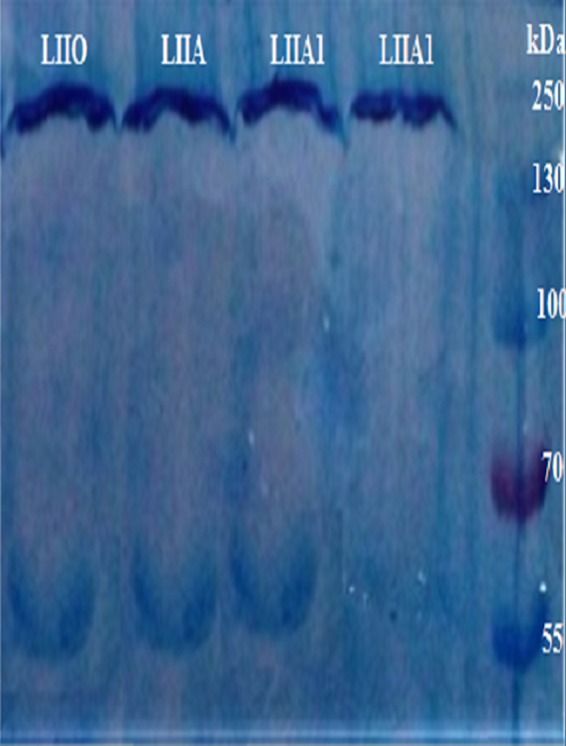
SDS-PAGE profiling of fragment antigen binding components following the digestion of anti-id antibodies (IgY) after staining with Coomassie brilliant blue for samples from groups LIIA1, LIIA, and LIIO.

The purified IgY antibodies, after fragmentation with pepsin was performed, proceeded to SDS-PAGE for the assessment of the Fab (fragment antigen binding) component. The sizes of the fragmented IgY proteins ranged between 55 to 70 kDa as represented in Fig. 1.

The presence of maximum Fab protein contents was recorded in group LIIA (serotype A), while group LIIA1 (serotype Asia 1) showed the second highest Fab protein concentrations as represented in Fig. 1. The density bands indicated that the third highest Fab protein contents were seen in group LIIO (serotype O) as measured through SDS-PAGE and depicted in Fig. 1.

### Sterility and safety studies of anti-id antigen suspensions.

The culture media inoculated with a suspension of monovalent Montanide adjuvanted anti-id FMD virus antigen (MATM) and trivalent Montanide adjuvanted anti-id FMD virus antigen (MATC) for all three serotypes (Asia 1, A, and O) indicated sterile preparations at up to 7 days postincubation, and no turbidity was recorded in the culture broth. Furthermore, the inoculations on the blood agar plates were also negative for any bacterial growth.

The change in body temperature of the inoculated rabbits was normal (101 ± 0.5°F to 103 ± 0.5°F), and no mortality was recorded. The other parameters were in normal range, with signs of mild swelling at the site of injections. The swelling from the inoculations was resolved after 24 h.

### Immune response to Montanide adjuvanted monovalent anti-id FMD virus antigens in mice.

**FIG 2:**
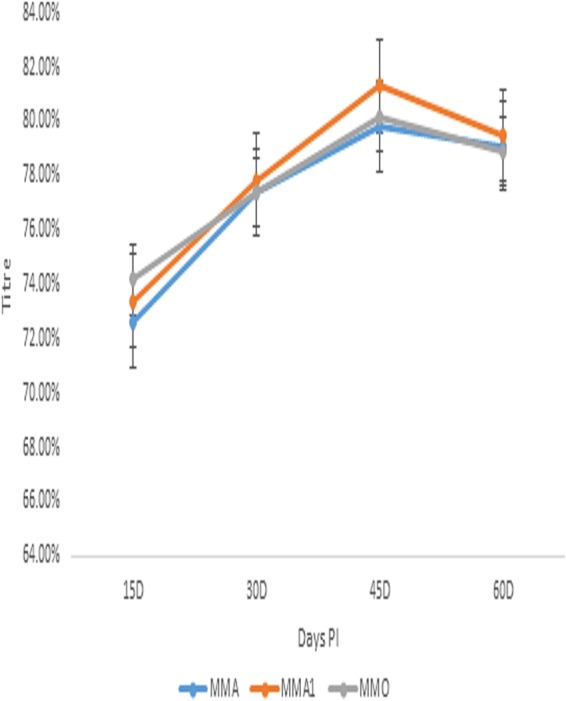
Immune response to Montanide adjuvanted monovalent anti-idiotype FMD virus antigens in mice.

The Montanide adjuvanted monovalent anti-id FMD virus antigen (MATM) performed similarly for all three FMD serotypes. The mean Ab titer of Montanide adjuvanted anti-id FMD virus antigen was initially low, but, over the course of the study, the Ab titer increased until day 45 p.i. The titer started decreasing at day 45 p.i.; the decline was slow as presented in Fig. 2. The mean Ab titer of the Montanide adjuvanted monovalent anti-id FMD virus antigen of serotype A (MMA) group remained in the range of 72.60% to 79.10% from day 15 p.i. to day 60 p.i., respectively. The initial mean antibody (Ab) titer was 72.60% at day 15 p.i. and increased to a maximum value of 79.80% at day 45 p.i. The titer remained stable, with a very minute decline to 79.10% recorded at day 60 p.i. as indicated in Fig. 2.

Group MMA of serotype Asia 1 (MMA1) yielded a variable range of mean Ab titers: initially, a titer of 73.40% was measured at day 15 p.i. followed by a stable rise in the mean Ab titer to a maximum of 81.30% at day 45 p.i. A slow decrease in the mean Ab titer to 79.50% at day 60 p.i. was observed as depicted in Fig. 2. A mean Ab titer of 74.20% for group MMA serotype O (MMO) was recorded at day 15 p.i.; the titer reached its maximum value of 80.20% at day 45 p.i. followed by a decline. At day 60 p.i., the mean Ab titer was 78.90%.

### Immune response to Montanide adjuvanted trivalent anti-id FMD virus antigen (MATM) in mice.

**FIG 3:**
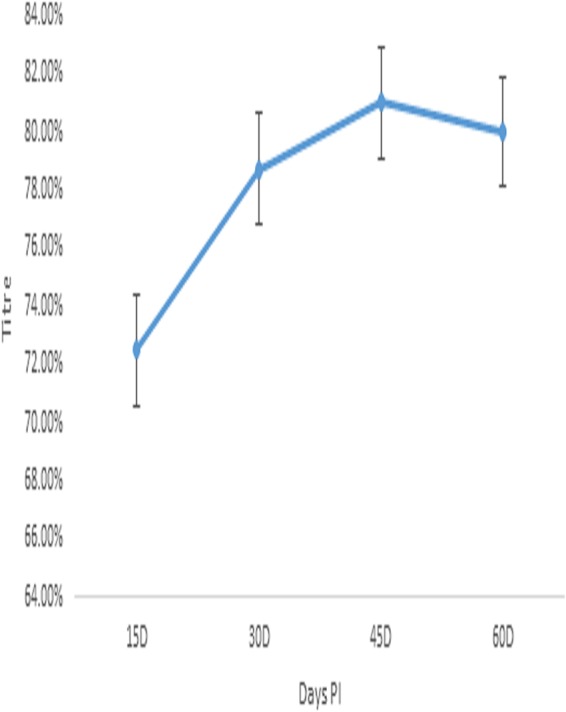
Immune response to Montanide adjuvanted trivalent anti-idiotype FMD virus antigen in mice.

A steady increase in the mean Ab titer for mice immunized against Montanide adjuvanted trivalent anti-id FMD virus antigen was recorded. The mean Ab titer was 72.50% at day 15 p.i. followed by a stable rise to 81% at day 45 p.i. The mean Ab titer decreased to 80% at day 60 p.i.; the decline in the mean Ab titer was slow, as presented in Fig. 3.

### Immune response to Montanide adjuvanted trivalent anti-id FMD virus antigen and Montanide adjuvanted trivalent FMD commercial vaccine in mice.

**FIG 4:**
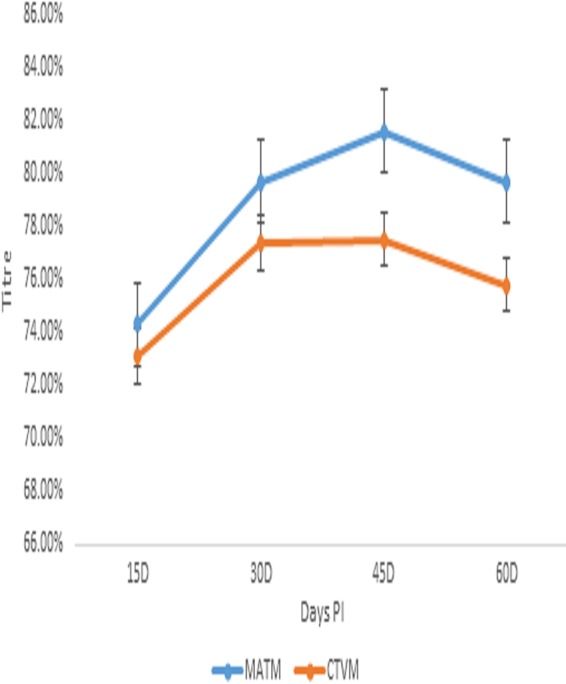
Comparative immune response to Montanide adjuvanted trivalent anti-idiotype FMD virus antigen and commercial trivalent FMD vaccine in mice.

The immune status of Montanide adjuvanted trivalent anti-id FMD virus antigen (MATM) was compared to the commercially available Montanide adjuvanted trivalent FMD vaccine (CTVM). The mean Ab titer of MATM ranged from 74.30% to 79.70% compared to the mean Ab titer range of 73.10% to 75.80% for CTVM as described for Fig. 4.

The mean Ab titer of MATM was 74.30% at day 15 p.i., which represented a nonsignificant difference from the 73.10% Ab titer of CTVM. A factorial increase in the Ab titer (79.70%) of MATM was observed that was significantly different from the 77.40% Ab titer of CTVM at day 30 p.i. The Ab titer of MATM persistently increased and reached its maximum value of 81.60% at day 45 p.i., whereas that of CTVM remained constant at 77.50%. The differences in the mean Ab titers of MATM and CTVM were significant at day 45 p.i.

A decline in the mean Ab titers of MATM and CTVM was recorded at day 60 p.i.; the mean Ab titer of MATM decreased to 79.70%, which was significantly different from the 75.80% mean Ab titer of CTVM at day 60 p.i. as depicted in Fig. 4.

### Immune response to Montanide adjuvanted trivalent anti-id FMD virus antigen and Montanide adjuvanted trivalent FMD commercial vaccine in calves.

The immune response to Montanide adjuvanted trivalent anti-id FMD virus antigen (MATC) was observed in calves.

**FIG 5:**
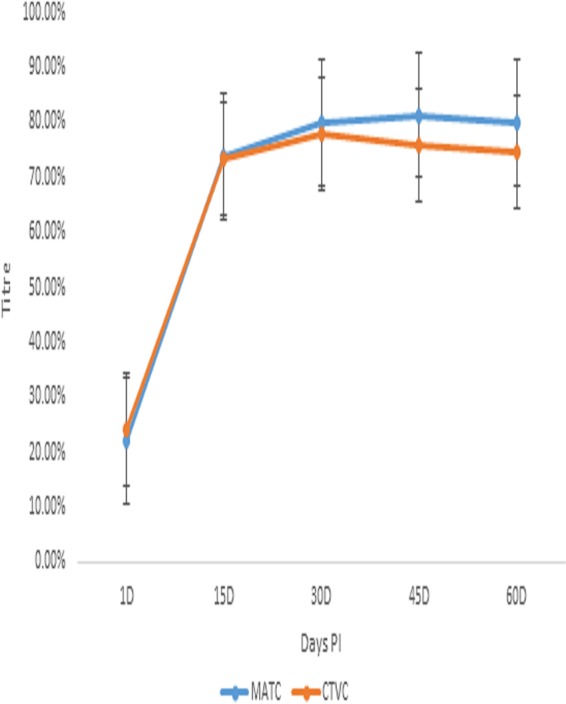
Comparative postimmunization antibody titers of Montanide adjuvanted trivalent anti-idiotype FMD virus antigen and Montanide adjuvanted trivalent FMD commercial vaccine in calves.

The Ab titer of Montanide adjuvanted trivalent FMD vaccine (CTVC) (commercially available) remained in the range of 71% to 79% from day 15 to day 60 p.i. The Ab titer increased to its maximum value up to day 30 p.i. followed by a continuous decrease as presented in Fig. 5.

The mean Ab titer of MATC steadily increased to 81.33% at day 45 p.i. followed by a slow decline to 80.00% at day 60 p.i. The mean Ab titer of CTVC was significantly different from that of MATC at day 45 and day 60 p.i. The mean Ab titer of CTVC continuously decreased from 76.00% to 74.66% from day 45 to day 60 p.i., respectively, as depicted in Fig. 5.

## DISCUSSION

The present study was performed to explore the potential of Ab2 as a surrogate viral antigen against FMD virus. The anti-id antigen was developed against three serotypes of FMD virus: serotypes A, Asia 1, and O. Anti-id antigen were generated from egg yolk antibodies. A study comparing responses to commercially available Montanide adjuvanted trivalent FMD vaccine and Montanide adjuvanted trivalent anti-id FMD virus antigen was performed in laboratory animals.

Ab2 was separated from the egg yolk of layer birds (injected with idiotype FMD virus antigen) with AS precipitation. The AS precipitation yielded up to 17 mg/ml of anti-id antibodies. The results of anti-id separation were similar to the results reported by Ko and Ahn, who demonstrated that the AS precipitation method is superior to cation exchange chromatography in terms of yield and purity for separation of antibodies (10). The use of AS precipitation alone results in better yield and purity than the use of octanoic acid for separation of IgY from the egg yolk (11). The results of the present study were correlated with those reported by Barati et al., who used the freeze-thaw method with the sodium chloride precipitation method for the purification of IgY from the egg yolk (12). IgY has a protective effect on the animal models and can be easily used for experimentation. The purified IgY was digested with pepsin to generate a Fab component. The pepsin digestion yielded Fab fragments of more than 50 kDa, in contrast to the results reported by Akita and Nakai, where the IgY fragmentation yielded Fab fragments of 44 kDa (13, 34).

The recent development of Ab1 and Ab2 as vaccines against bacterial and viral diseases is a great achievement (9). Anti-id vaccines give a protective immune response against certain cancers where the immune system could not recognize self-antigens (14). Yildirim et al. demonstrated that the use of anti-id vaccine against hepatitis B cell surface antigens provided protective immunity to infectious doses (15). However, this protective immunity is genetically restricted in humans, as the species represents an outbred population. Sacks et al. reported similar induction of immune responses in the absence of antigen using an Ab2 against African trypanosomiasis (16). In the present research, Ab2 was used as a surrogate antigen to induce an immune response against FMD virus. As discussed earlier, only β Ab2 has the potential to bind the paratope of Ab1 to mimic the molecular structure of original antigenic epitope. The polyclonal Ab1 used to generate Ab2 contains not only β but also all other types of Ab2. The non-paratope-specific Ab2 may not block by viral attachment except for nonspecific stearic hindrance. As evidenced by previous studies by Chiang and Nicholson, a 60% level of inhibition of viral attachment suggests a high percentage of β Ab2 mimicking the viral antigen (17). In the present study, the range of mean Ab titer recorded was 73% to 81%, which correlates with results reported from previous studies, as only the β Ab2 can mimic viral antigens to give Ab titers. Thus, the Ab2 generated was against Fab Ab1. Rimmelzwaan et al. classified the Ab2 as Ab2β or Ab2γ on the basis of binding to the idiotype antigen and inhibition of binding by the antigen (18). Previously, anti-id neutralizing antibodies were generated against many animal diseases caused by, e.g., rabies virus, herpes simplex virus, poliovirus, etc., to modulate the immune responses (19–22) and raised anti-anti-id antibodies (Ab3) that reacted only against the anti-id immunogen rather than against the original viral antigen to elicit an immune response.

A neutralizing effect of Ab2 was recently reported against Dengue virus (DENV). Ab2 was raised against potent human DENV serotype 1-specific antibodies named HM14c10; structural mimicry by Ab2 was achieved after several interactions with the heavy chain of antibodies to neutralize the DENV (23). A neutralizing effect of monoclonal Ab2 against reovirus of the *Picornaviridae* family was reported by Sharpe et al. (24). The manipulation of the immune responses of both B and T cells was recorded in the absence of adjuvant. It was reported that Ab2 blocks viral attachment to the cell and enhances cytotoxic T lymphocyte-dependent lysis of infected cells in a dose-dependent manner. In the current study, the immune response to Montanide adjuvanted monovalent and trivalent anti-id FMD virus antigens was evaluated. The Montanide adjuvanted monovalent and trivalent anti-id FMD virus antigens produced Ab titers ranging from 70% to 83%. *In vitro* experimentation was done for the preparation of monoclonal antibodies and anti-id in mouse model as described previously by Garmendia et al. (25). The Ab2 was used as a surrogate antigen for FMD virus. Mice were immunized by injecting Ab2 and anti-anti-id (Ab3), and Ab2 showed a higher response than Ab3 in the mouse model. Paryati et al. compared rabies virus vaccines with oral and intramuscular (i.m.) suspensions of the anti-id vaccine. The enzyme-linked immunosorbent assay (ELISA) Ab titer of i.m. anti-id vaccines was higher than that of the oral suspension (26). In the present experiments, an anti-id antigen suspension was injected for comparison to the trivalent FMD viral vaccine. The results of Ab titration support the hypothesis of the use of Ab2 as a surrogate antigen. A mean Ab titer range of 72% to 83% was observed in the mice injected with polyclonal anti-id antigen prepared from the IgY antibodies. The initial response in Montanide adjuvanted anti-id FMD virus antigen was slow and increased over time to 83% followed by a slow decline. The results were correlated with those from the experiments of Weiyun et al. (27). The overall survival rate of patients receiving chemotherapy against follicular lymphoma was determined. Ab2 was associated with superior overall survival in the patients with follicular lymphoma. In the current study, Montanide adjuvanted inactivated FMD virus injected into goats produced a maximum Ab1 titer of 87%, whereas Montanide adjuvanted anti-id antigen produced a maximum titer of 83% in the mice examined. Administration of the commercial trivalent Montanide adjuvanted FMD killed-virus vaccine resulted in a mean Ab titer of 77.50% in mice at day 45 p.i. However, Montanide adjuvanted trivalent anti-id antigen produced a persistent titer of 81% at day 60 p.i. The commercially available inactivated vaccines have the potential to alter the functional epitopes of the virus and may lead to the development of permanently nonneutralizing antibodies in the animals (28). The mean Ab titer that was determined in the present study correlated with the results of the previous similar study by Handajani et al., who performed comparative analysis of three parameters through least-signficant-difference testing (29). The results are also supported by the results of the previous trial of Arif et al. (8), where Ab2 was developed against outer membrane protein of Pasteurella multocida. The immune potentiating effects were observed in the rabbits. Persistent immunity was recorded with OMP Ab2 compared to plain bacitracin, with the results giving evidence of a better immunogenic response by anti-id antibodies. The anti-id-based vaccines may be able to balance the immune response through antigenic mimicry and provide a prophylactic immune response to the virus (30). In the current study, the mean Ab titer of Montanide adjuvanted trivalent FMD virus antigen was compared to that of Montanide adjuvanted trivalent FMD vaccine in the natural host. The mean Ab titer of Montanide adjuvanted trivalent FMD vaccine ranged 73.66% to 78.00%. The maximum mean Ab titer was recorded at day 45 p.i. and was followed by a decline to 74.66% at day 60 p.i. However, Montanide adjuvanted trivalent anti-id FMD virus antigen behaved differently. The maximum mean Ab titer of 81.33% was recorded at day 45 p.i. The decline in the Ab titer was slow, and a titer of 80.00% was observed at day 60 p.i. in calves. In the challenge/protection test, all the immunized animals showed 100% protection from the FMD virus. The strong and persistent immune response proved the potential of substitution of FMD virus with anti-id antigen in the vaccine. The results of the present study is also supported by data from the trials conducted by Levy el al. in which MyVax vaccine was compared with anti-id vaccine in patients with advanced follicular lymphoma. The anti-id vaccine generated outcomes superior to those seen with MyVax vaccine (31).

## MATERIALS AND METHODS

The idiotype antibodies (Ab1) against FMD virus were developed in experimental goats. Briefly, all three of serotypes A, Asia 1, and O of FMD virus were inactivated and injected into the goats through the intramuscular (i.m.) route for the preparation of polyclonal Ab1. Fab (fragment antigen binding) from the Ab1 was separated and reinjected into layer birds through the i.m. route for the development of anti-id antibodies (Ab2). The development of anti-id FMD antibodies in the egg yolks was confirmed through the use of the agar gel immunoprecipitation test (AGPT). Table 1 lists all the animals used in the study.

**TABLE 1:** Details of the animals with their grouping and purpose in the study

Animal category	Groups	Purpose
Goats		Idiotype antibody production
Layer birds	LIIA, LIIA1, and LIIO	Anti-idiotype antibody production
Rabbits		Safety studies
Mice	MMA1, MMA, MMO, MAT, UIC	Immune response of monovalent and trivalent anti-idiotype antigen
Mice	MATM, CTVM, UICM	Immune response of trivalent anti-idiotype antigen and commercial vaccine
Calves	MATC, CTVC, UICC	Immune response of trivalent anti-idiotype antigen and commercial vaccine

### Separation and purification of anti-id antibodies.

The eggs collected from layer birds previously injected with idiotype FMD virus antigen were processed separately for the purification of Ab2 against all three FMD serotypes (A, Asia 1, and O). IgY was separated through the use of the ammonium sulfate (AS) precipitation method as described by Ferella et al. (32). The purified Ab2 (IgY) collected from egg yolk were subjected to digestion for the separation of Fab components. Purified Fab was dialyzed against 50 volumes of phosphate-buffered saline with a pH of 7.0 (33). The specificity of the Ab2 Fab fragments of the three serotypes (A, Asia 1, and O) of FMD virus was confirmed through SDS-PAGE. The serotypes were marked LIIA, LIIA1, and LIIO, respectively.

### Preparation of Montanide adjuvanted monovalent and trivalent anti-id FMD virus antigen.

The total protein contents of Fab were adjusted to 10 mg/ml. The prepared Fab from Ab2 was emulsified in Montanide adjuvant (1:1) for the preparation of monovalent anti-id antigens separately for three serotypes (Asia 1, A, and O) of FMD, while a volume containing 10 mg/ml anti-id protein concentrations for each of the three serotypes (Asia 1, A, and O) was mixed at 1:1:1 to prepare a trivalent anti-id FMD virus antigen. The suspension was emulsified in Montanide adjuvant (1:1) and stored at <10°C until further use.

### Sterility and safety of Montanide adjuvanted monovalent and trivalent anti-id FMD virus antigen suspensions.

The prepared anti-id FMD virus antigens (monovalent and trivalent) for all three serotypes (A, Asia 1, and O) were studied to analyze their sterility and safety. Thioglycolate broth and blood agar media were inoculated with 1 ml of each suspension and observed at 37°C for 7 days. The rabbits were immunized subcutaneously (s.c.) in the neck region and observed for changes in body temperature and morbidity for up to 7 days postimmunization (p.i.) for safety studies.

### Immune response to Montanide adjuvanted monovalent and trivalent anti-id FMD virus antigens in mice.

The immunogenicity of Montanide adjuvanted monovalent and trivalent anti-id FMD virus antigens was analyzed in albino white mice (25 to 30 g body weight).

Fifty mice were divided into five groups (10 mice in each); the groups were named MMA1 (for “Montanide adjuvanted monovalent anti-id FMD virus antigen of serotype Asia 1”) and MMA and MMO (serotype A and O, respectively). Similarly, additional groups were named MAT (for “Montanide adjuvanted trivalent anti-id FMD virus antigen”) and UIC (for “uninoculated control group”). A 0.2-ml volume of monovalent anti-id FMD virus antigen was injected through the subcutaneous (s.c.) route in the members of groups MMA1, MMA, and MMO for serotype Asia 1, A, and O, respectively. A 0.2-ml volume of Montanide adjuvanted trivalent anti-id FMD virus antigen was injected into group MAT, while group UIC remained an uninoculated control group. A booster dose of 0.2 ml was injected after 2 weeks followed by the collection of serum samples at days 0, 15, 30, 45, and 60. The Ab titer was determined through the use of a competitive ELISA kit (ID Vet; Izsler, Brescia, Italy) according to the protocols of the manufacturer. A titer above 70% at a 1/10 dilution was considered to represent a positive result.

### Immune response to Montanide adjuvanted trivalent anti-id FMD virus antigen and commercial FMD vaccine in mice.

The immunogenicity of Montanide adjuvanted trivalent anti-id FMD virus antigen and commercial FMD vaccine was evaluated in albino white mice (25 to 30 g body weight).

Thirty mice were divided into three groups, namely, the MATM group (for “Montanide adjuvanted trivalent anti-id FMD virus antigen”), the CTVM group (for “commercial trivalent FMD vaccine”), and the UICM group (for “the uninoculated control group”) (10 mice in each group). A 0.2-ml volume of commercial FMD vaccine (Cell Culture trivalent Montanide adjuvant; FMD Research Center, Lahore, Pakistan) was injected s.c. in the members of the CTVM group, and 0.2 ml of Montanide adjuvanted trivalent anti-id FMD virus antigen was injected into members of the MATM group, while the members of the UICM group were kept as uninoculated controls. A booster dose of 0.2 ml was injected after 2 weeks followed by the collection of serum samples at days 0, 15, 30, 45, and 60. The Ab titer was determined through the use of a competitive ELISA kit.

### Immune response to Montanide adjuvanted trivalent anti-id FMD virus antigen and commercial FMD vaccine in calves.

For the experimental trials in calves, animals from the two volunteer farmers were managed at their own farm under conditions of good management. A total of nine animals were segregated into the following three groups: a MATC group (calves), a CTVC group (calves), and a UICC group (uninoculated controls). The members of group CTVC were injected s.c. with a single dose of 5 ml commercially available FMD killed-virus vaccine. A single dose of 5 ml Montanide adjuvanted trivalent anti-id FMD virus antigen was injected through the s.c. route in the members of the MATC group. Group UICC, with three animals, was treated as the uninoculated control group. A booster dose was given after 1 week, and blood samples were collected for serum separation. The titers of the antibodies were checked through the use of a competitive ELISA kit.

### Statistical analysis.

The results of the Ab titer determination experiments were evaluated statistically using factorial analysis. The comparative mean Ab titers were measured through the use of Fisher’s least-significant-difference test.
